# Draft Genome Sequence of the Cellulolytic and Xylanolytic Thermophile *Clostridium clariflavum* Strain 4-2a

**DOI:** 10.1128/genomeA.00797-15

**Published:** 2015-07-23

**Authors:** Elise A. Rooney, Kenneth T. Rowe, Anna Guseva, Marcel Huntemann, James K. Han, Amy Chen, Nikos C. Kyrpides, Konstantinos Mavromatis, Victor M. Markowitz, Krishna Palaniappan, Natalia Ivanova, Amrita Pati, Konstantinos Liolios, Henrik P. Nordberg, Michael N. Cantor, Susan X. Hua, Nicole Shapiro, Tanja Woyke, Lee R. Lynd, Javier A. Izquierdo

**Affiliations:** a Department of Biology, Hofstra University, Hempstead, New York, USA; b Thayer School of Engineering, Dartmouth College, Hanover, New Hampshire, USA; c BioEnergy Science Center, Oak Ridge National Laboratory, Oak Ridge, Tennessee, USA; d DOE Joint Genome Institute, Walnut Creek, California, USA

## Abstract

*Clostridium clariflavum* strain 4-2a, a novel strain isolated from a thermophilic biocompost pile, has demonstrated an extensive capability to utilize both cellulose and hemicellulose under thermophilic anaerobic conditions. Here, we report the draft genome of this strain.

## GENOME ANNOUNCEMENT

The ability of thermophilic clostridia to extensively degrade lignocellulosic materials and produce fermentation products that may serve as biocommodities makes them excellent candidates for consolidating bioprocessing applications (1). The genome sequence of *Clostridium clariflavum* DSM 19732 revealed novel mechanisms among thermophilic clostridia to break down cellulose and hemicellulose (2). *C. clariflavum* strain 4-2a is a novel strain isolated from compost (3), which has demonstrated the additional ability to utilize xylose and considerably degrade unpretreated switchgrass (4).

Genomic DNA of *C. clariflavum* strain 4-2a was obtained through a phenol-chloroform-CTAB extraction procedure, as previously described (2). The draft genome was generated at the DOE Joint Genome Institute (JGI) using Illumina data (5) from a short-insert paired-end library with an average insert size of 270 bp, generating 24,015,970 reads, and an Illumina long-insert paired-end library with an average insert size of 6,484.62 bp, generating 25,583,980 reads and totaling 7,440 Mbp of Illumina data (F. Chen, personal communication).

Protocols for library construction and sequencing performed at the JGI can be found at http://www.jgi.doe.gov. The initial draft data was assembled with ALLPATHS version 39750 (6) and contained 156 contigs in 18 scaffolds, and the consensus was shredded into 10-kbp overlapping shreds. The Illumina draft data were assembled with Velvet version 1.1.05 (7), and consensus sequences were computationally shredded into 1.5-kbp overlapping shreds. The Illumina draft data were assembled again with Velvet using shreds from the first Velvet assembly to guide the next assembly. The consensus from the second Velvet assembly was shredded into 1.5-kbp overlapping shreds. The shreds from the ALLPATHS and Velvet assemblies and a subset of Illumina CLIP paired-end reads were assembled using parallel Phrap version 4.24 (High Performance Software, LLC). Misassemblies were corrected in Consed (8–10). Gap closure was accomplished using repeat resolution software (W. Gu, personal communication) and sequencing of 104 PCR PacBio consensus sequences (C. Han, personal communication). The total size of the genome is 4.9 Mb and the final assembly is based on 7,440 Mbp of Illumina data, providing 1,518× coverage. Project information is available in the Genomes Online Database (11), and the final annotation is available from the Integrated Microbial Genome (IMG) system (12). Genes were identified using Prodigal (13) and GenePRIMP (14) as part of the JGI’s microbial annotation pipeline (15). Additional gene prediction and functional annotation was performed with the IMG-ER platform (16).

Initial findings from the genome of *C. clariflavum* strain 4-2a include the presence of genes coding for xylose isomerase and xylulose kinase in a genomic island not present in the type strain, DSM 19732. The inventory of glycoside hydrolases is almost identical to the one reported for the type strain (2), except for an additional beta-glucosidase/xylosidase from glycoside hydrolase family 3 identified in the same genomic island. Further ongoing analysis is expected to provide insight into this strain’s lignocellulolytic capabilities and inform the development of genetic tools.

### Nucleotide sequence accession numbers.

The genome sequence of *C. clariflavum* 4-2a is deposited in GenBank under the accession numbers ASAA01000001 to ASAA01000016 and the annotated genome in IMG through accession number 2524614797.
